# 引言

**DOI:** 10.3724/SP.J.1123.2022.12016

**Published:** 2023-02-08

**Authors:** Jingwu KANG, Tongdan WANG

药物分析一直是色谱技术最重要的应用领域之一。色谱技术几乎贯穿了药物研发的整个流程，无论从早期的靶标鉴定、先导分子筛选，还是工艺研发、产品质量研究与控制、杂质分析与鉴定、药理药代及临床样品分析等环节，其均起着不可或缺的作用。

近年来，无论是在传统的化学制药，还是新兴的生物医药，中国制药界都展露出突飞猛进的发展势头。为了进一步促进国内科研学术界和制药工业界从事药物色谱分析人员的深度技术交流与广泛合作，受《色谱》期刊委托，我们组织了一期“药物分析”专辑，诚挚邀请了国内相关高等院校、科研院所、行政单位及工业界分析工作者为本刊撰稿。

本专辑由1篇专论与综述、7篇研究论文和2篇技术与应用组成，涉及肝素结构色谱分析研究进展、消毒剂及护理品分析、人全血中磷脂酰乙醇普查、酪氨酸激酶抑制剂分析、中药的定量指纹图谱分析、手性分离等。我们衷心希望通过本专辑的出版，为色谱分析技术在药物分析界的广泛应用抛砖引玉，加速中国制药水平的攀升步伐，为促进人民普惠健康事业和国民经济的发展做出贡献。

虽然经受了疫情的影响，但各地科研工作者仍夜以继日的坚守一线，大胆探索，攻克各类技术难题，“药物分析”专辑最终在大家的不懈努力下出版，实属不易。衷心感谢各位作者及审稿专家的鼎力支持和奉献！

本专辑客座主编

中国科学院上海有机化学研究所 上海药明生物技术有限公司

康经武 研究员 汪彤丹


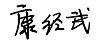
 
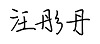


2022年12月12日

